# Logistic Regression Model for a Bivariate Binomial Distribution with Applications in Baseball Data Analysis

**DOI:** 10.3390/e24081138

**Published:** 2022-08-17

**Authors:** Yewon Han, Jaeho Kim, Hon Keung Tony Ng, Seong W. Kim

**Affiliations:** 1 Department of Applied Mathematics, Hanyang University, Ansan 15588, Korea; 2 Department of Economics, Hanyang University, Ansan 15588, Korea; 3 Department of Mathematical Sciences, Bentley University, Waltham, MA 02452, USA

**Keywords:** bivariate binomial distribution, gibbs sampling, logistic regression, Metropolis–Hastings algorithm, random effect, posterior mean, 62J05, 62F15, 60E05, 62H10

## Abstract

There has been a considerable amount of literature on binomial regression models that utilize well-known link functions, such as logistic, probit, and complementary log-log functions. The conventional binomial model is focused only on a single parameter representing one probability of success. However, we often encounter data for which two different success probabilities are of interest simultaneously. For instance, there are several offensive measures in baseball to predict the future performance of batters. Under these circumstances, it would be meaningful to consider more than one success probability. In this article, we employ a bivariate binomial distribution that possesses two success probabilities to conduct a regression analysis with random effects being incorporated under a Bayesian framework. Major League Baseball data are analyzed to demonstrate our methodologies. Extensive simulation studies are conducted to investigate model performances.

## 1. Introduction

There has been a considerable amount of research work on modeling offensive and defensive abilities in baseball. In particular, offensive measures are often assessed and analyzed to predict future hitting performance. Further, various measures have been proposed in the past decades through Sabermetrics (known initially as SABRmetrics) to evaluate batters’ hitting performance. As mentioned by [1], successful free-agent hitters who are expected to produce similar future outcomes are entitled to an enormous amount of money with large contracts. To estimate the parameter of interest, *the probability of success* (e.g., the probability of hits or the batting probability of a baseball player), in this scenario, the binomial regression model is a commonly used model with a generic term in conjunction with regression covariates.

Many studies have been conducted on binomial regression models with well-known link functions such as the logistic, probit, and complementary log-log functions. Ref. [2] discussed hierarchical models for analyzing binomial data in a Bayesian framework. Bedrick et al. [3] presented extensive Bayesian methodologies for binomial regression models without incorporating random effects. Later, Chenetal [4] investigated the theoretical properties of the Jeffreys prior to general binomial regression models. Pires and Diniz [5] and Prasetyo et al. [6] also analyzed binomial regression models based on the Bayesian perspective. Here, we notice that the conventional binomial regression model is applicable only when a single success probability is associated with players or teams. However, we often encounter situations where two dependent probabilities of success are of interest when analyzing the offensive sides of hitters in baseball. Specifically, the ‘batting average’ is an older well-recognized measure to evaluate batters’ performance. In the modern baseball era, the ‘slugging percentage’ is another crucial measure to see how often power hitters can produce extra-base hits [7]. Under these circumstances, it is important to investigate which covariates are related to these two dependent measures separately and simultaneously.

The bivariate binomial (BVB) distribution originally proposed by [8] is one of the remedies for dealing with two success probabilities when nested binary data come into play. Based on the BVB distribution, several theoretical properties, including Jeffreys priors, were investigated by [9]. Ref. [10] suggested a bivariate extension of the binomial autoregressive (AR) model and proposed a new class of bivariate binomial AR models based on a binomial thinning operation. Recently, Ref. [11] conducted a changepoint analysis with the BVB to investigate the streakiness of baseball and basketball players. However, to the best of our knowledge, a regression model for the BVB distribution has not yet been studied in the literature.

In this paper, we utilize the BVB distribution with two inter-linked logit functions to capture the association between two success probabilities based on nested binary data. The proposed model allows the two inter-linked logit functions to share some common covariates. In other words, some covariates are included in both logit functions and some covariates are included in only one of the logit functions. Another novel feature of our proposed model is that unobserved characteristics of individuals or groups that simultaneously affect two success probabilities are taken into account via joint random effects. The joint random effects are unobserved heterogeneity that commonly exists in both dependent success probabilities. We treat the joint random effects as model parameters and estimate them using a Markov Chain Monte Carlo (MCMC) algorithm under the Bayesian paradigm. The direct estimation of the joint random effects is important for two reasons. First, in a regression analysis setting, random effects typically represent subject-specific means in a longitudinal dataset that are assumed to be independent of any covariates. Those random effects are treated as an additional source of regression errors. However, as in our empirical application, the random effects contain valuable information about individuals or groups and can be useful for prediction. For instance, the average performance over the full sample period can differ significantly across batters, and it is likely that these differences among players will persist in the future. Estimating these random effects directly will provide a way to extract useful subject-specific information. Second, by directly estimating the common random effects, we can test if the unobserved heterogeneity of one success probability is associated with its counterpart in the pair of success probabilities. In addition, our direct estimation approach provides empirical results that are robust to potential non-zero correlations between unobserved heterogeneity and the covariates in the two logit functions. When the unobserved heterogeneity is correlated with the covariates in a regression model, it is typically modeled with fixed effects and the unobserved heterogeneity should be explicitly estimated or eliminated before model fitting by data transformations such as differentiating successive observations or demeaning observations by their time-series means. However, these data transformations are difficult to apply when using a logit function. In contrast, our proposed approach can handle the non-zero correlations between covariates and the unobserved heterogeneity through a direct estimation method.

In this work, we consider the Bayesian approach for the parameter estimation, since the Bayesian method is more suitable for the estimation in the proposed model than the classical maximum likelihood (ML) estimation method because high-dimensional parameter spaces are involved in the model. Due to the longitudinal nature of data, the number of model parameters, including individual random effects, increases linearly with the cross-section size. The high-dimensional parameter space involved in the model brings additional difficulties to the parameter estimation process for the ML method. For instance, the ML estimates are mostly not in closed-form and require a numerical method to solve the optimization problem. For high-dimensional parameter spaces, obtaining a reasonable set of initial estimates of the model parameters for the ML estimation method can be challenging. Subsequently, the chance of locating a local maximum instead of the global maxima in the numerical optimization process can be higher. Moreover, the numerical optimization algorithm involved in the ML method could be very unstable when the sample size is small in the time dimension. In application to the baseball data analysis, we consider a panel dataset with T=6 (6 months) with N=60 (60 players). The reason we choose T=6 is because that we want the player performances to be as homogeneous as possible during the sample period, and that a single regular season in Major League Baseball (MLB) consists of about six months. Typically, a baseball player’s performance varies significantly from season to season. Therefore, restricting the sample period to one season keeps the homogeneity of the data and allows us to model the average performance of each player using the random effect. However, the relatively small sample may cause difficulties in the maximum likelihood estimation for the time series dimension. These technical issues in the estimation process can be readily avoided using the Bayesian estimation method by suitably imposing Bayesian priors over the model parameters.

The rest of this paper is organized as follows. In Section 2, we introduce the mathematical notations and the BVB distribution along with the three proposed BVB regression models. Section 3 discusses the Bayesian inference based on the proposed BVB regression models and provides the Bayesian MCMC algorithm. A practical data analysis based on the performance data of MLB players is presented in Section 4. Then, to evaluate the performance of the proposed models and estimation methods, Monte Carlo simulation studies are used and the settings and results are presented in Section 5. Finally, brief concluding remarks along with future research directions are provided in Section 6.

## 2. Models and Notations

We use $D_{\ell }=\left\{\left(m_{\ell 1},x_{\ell 11},x_{\ell 21},z_{\ell 1}\right),\left(m_{\ell 2},x_{\ell 12},x_{\ell 22},z_{\ell 2}\right),\ldots ,\left(m_{\ell T_{\ell }},x_{\ell 1T_{\ell }},x_{\ell 2T_{\ell }},z_{\ell T_{\ell }}\right)\right\}$ to denote the observed sequence of nested bivariate binary data for the *ℓ*-th individual/group (ℓ=1,2,…,L) at time points $t=1,2,\ldots ,T_{\ell }$ with the covariate vector $z_{\ell t}=\left(1,z_{\ell t1},z_{\ell t2},\ldots ,z_{\ell tK}\right)$ that contains *K* covariates, where the bivariate random vector $\left(x_{\ell 1t},x_{\ell 2t}\right)$ with $x_{\ell 1t}\geq x_{\ell 2t}$ follows a BVB distribution denoted by

$$\begin{array}{c} \left(x_{\ell 1t},x_{\ell 2t}\right)∼BVB\left(m_{\ell t},p_{\ell t},q_{\ell t}\right) \end{array}$$

for $t=1,2,\ldots ,T_{\ell }$. The BVB distribution considered here is based on a two-stage binomial model. For example, in assessing the performance of a baseball player, say the *ℓ*-th player at time *t*, suppose the probability of hits is $p_{\ell }$, then the number of hits out of $m_{\ell t}$ total at-bats is a random variable $X_{\ell 1t}$ that follows a binomial distribution

$$\mathrm{Pr}\left(X_{\ell 1t}=x_{\ell 1t}|m_{\ell t}\right)=\left(\begin{array}{c} m_{\ell t}\\ x_{\ell 1t} \end{array}\right)p_{\ell t}^{x_{\ell 1t}}{\left(1-p_{\ell t}\right)}^{m_{lt}-x_{\ell 1t}},$$

$x_{\ell 1t}=0,1,\ldots ,m_{\ell t}$. For the same player at time *t*, suppose the probability of extra-base hits (two-base hits, three-base hits, and home runs) out of total hits is $q_{\ell t}$. Given the number of hits $X_{\ell 1t}=x_{\ell 1t}$, the number of extra-base hits is a random variable $X_{\ell 2t}$ that follows a binomial distribution

$$\begin{array}{c} \mathrm{Pr}\left(X_{\ell 2t}=x_{\ell 2t}|m_{\ell t},x_{\ell 1t}\right)=\left(\begin{array}{c} x_{1\ell t}\\ x_{\ell 2t} \end{array}\right)q_{\ell t}^{x_{\ell 2t}}{\left(1-q_{\ell t}\right)}^{x_{l\ell t}-x_{\ell 2t}}, \end{array}$$

$x_{\ell 2t}=0,1,\ldots ,x_{\ell 1t}$. From [8], the joint probability mass function of $x_{\ell 1t}\geq x_{\ell 2t}$ that follows the BVB distribution is given by

$$\begin{array}{ccc} f(x_{\ell 1t},x_{\ell 2t};m_{\ell t},p_{\ell t},q_{\ell t}) & = & \left(\begin{array}{c} m_{\ell t}\\ x_{\ell 1t} \end{array}\right)p_{\ell t}^{x_{\ell 1t}}{\left(1-p_{\ell t}\right)}^{m_{lt}-x_{\ell 1t}}\left(\begin{array}{c} x_{\ell 1t}\\ x_{\ell 2t} \end{array}\right)q_{\ell t}^{x_{\ell 2t}}{\left(1-q_{\ell t}\right)}^{x_{\ell 1t}-x_{\ell 2t}},\\ & & \;x_{\ell 1t}=0,1,\ldots ,m_{\ell t},x_{\ell 2t}=0,1,\ldots ,x_{\ell 1t},\\ & & \;0<p_{\ell t}<1,0<q_{\ell t}<1. \end{array}$$

Here, $x_{\ell 1t}$ is the number of successes out of $m_{\ell t}$ trials with the probability of success $p_{\ell t}$ and $x_{\ell 2t}$ is the number of successes out of $x_{\ell 1t}$ trials with probability of success $q_{\ell t}$.

**Remark** **1.** *The motivation of our work is based on nested (two-stage) binary data encountered in basketball and baseball, as well as in other fields such as microbiology [8]. The basketball data analyzed in [11] have a similar structure in which m is the number of shoot attempts, $x_{1}$ is the number of shoots made out of the m attempts, and $x_{2}$ is the number of three-point field goals out of the $x_{1}$ shots made. Following the same idea, one can construct a trivariate binomial distribution based on a three-stage binomial model by incorporating another nested stage. For example, in the basketball example, $x_{3}$ can be the number of 4-point plays (i.e., a player completes a three-pointer while being fouled, which leads to a free-throw) out of the $x_{2}$ three-point field goals.*

Wallis [12] proposed the logit transformation $\mathrm{logit}\left(p_{\ell t}\right)=\log\left(p_{\ell t}/\left(1-p_{\ell t}\right)\right)$ as an appropriate transformation for percentages, proportions, and probabilities because the logit transformation stabilizes the variance and brings the range of the data from (0,1) to (−∞,∞), which improves the performance of normal approximation. Moreover, the logit transformation ensures that the estimates and projections are in the interval (0,1). Since we have two inter-related success probabilities $p_{\ell t}$ and $q_{\ell t}$, we consider the following three logistic regression models based on the BVB distribution that can be formulated as follows.

- Model 0—Model with no random effect
  We consider the following two logit link functions to model the two inter-related success probabilities $p_{\ell t}$ and $q_{\ell t}$ with the covariates:
  (1) $$\begin{array}{ccc} \mathrm{logit}(p_{\ell t}) & = & \beta_{p0}^{\left(0\right)}+\beta_{p1}^{\left(0\right)}z_{\ell t1}+\ldots +\beta_{pk}^{\left(0\right)}z_{\ell tK}=z_{\ell t}\beta_{p}^{{}^{\prime }\left(0\right)},\\ \mathrm{logit}(q_{\ell t}) & = & \beta_{q0}^{\left(0\right)}+\beta_{q1}^{\left(0\right)}z_{\ell t1}+\ldots +\beta_{qk}^{\left(0\right)}z_{\ell tK}=z_{\ell t}\beta_{q}^{{}^{\prime }\left(0\right)}, \end{array}$$
  for ℓ=1,2,…,L, where $\beta_{p}^{\left(0\right)}=\left(\beta_{p0}^{\left(0\right)},\beta_{p1}^{\left(0\right)},\beta_{p2}^{\left(0\right)},\ldots ,\beta_{pK}^{\left(0\right)}\right)$ and $\beta_{q}^{\left(0\right)}=\left(\beta_{q0}^{\left(0\right)},\beta_{q1}^{\left(0\right)},\beta_{q2}^{\left(0\right)},\ldots ,\beta_{qK}^{\left(0\right)}\right)$ are the vectors of regression coefficients associated with parameters $p_{\ell t}$ and $q_{\ell t}$, respectively. The model in Equation (1) is our benchmark model and hence, we refer it to as *Model 0*.
- Model 1—Model with joint random effect
  We consider the following two logit link functions to model two inter-related success probabilities $p_{\ell t}$ and $q_{\ell t}$ with the covariates:
  (2) $$\begin{array}{ccc} \mathrm{logit}(p_{\ell t}) & = & \beta_{p0}^{\left(1\right)}+\beta_{p1}^{\left(1\right)}z_{\ell t1}+\ldots +\beta_{pk}^{\left(1\right)}z_{\ell tK}+a_{\ell }^{\left(1\right)}=z_{\ell t}\beta_{p}^{{}^{\prime }\left(1\right)}+a_{\ell }^{\left(1\right)},\\ \mathrm{logit}(q_{\ell t}) & = & \beta_{q0}^{\left(1\right)}+\beta_{q1}^{\left(1\right)}z_{\ell t1}+\ldots +\beta_{qk}^{\left(1\right)}z_{\ell tK}+\beta^{*(1)}a_{\ell }^{\left(1\right)}\\ & = & z_{\ell t}\beta_{q}^{{}^{\prime }\left(1\right)}+\beta^{*(1)}a_{\ell }^{\left(1\right)}, \end{array}$$
  for ℓ=1,2,…,L, where $\beta_{p}^{\left(1\right)}=\left(\beta_{p0}^{\left(1\right)},\beta_{p1}^{\left(1\right)},\beta_{p2}^{\left(1\right)},\ldots ,\beta_{pK}^{\left(1\right)}\right)$ and $\beta_{q}^{\left(1\right)}=\left(\beta_{q0}^{\left(1\right)},\beta_{q1}^{\left(1\right)},\beta_{q2}^{\left(1\right)},\ldots ,\beta_{qK}^{\left(1\right)}\right)$ are the vectors of regression coefficients associated with parameters $p_{\ell t}$ and $q_{\ell t}$. Here, we assume that $a_{1}^{\left(1\right)}$, $a_{2}^{\left(1\right)}$, $\ldots ,a_{L}^{\left(1\right)}$ are independent and identically distributed standard normal random variables representing a random effect, and parameter $\beta^{*(1)}$ indicates a varying deviance between subjects or individuals. The model in Equation (2) incorporates the joint random effects in the two logit functions. The key model parameter, $\beta^{*(1)}$, captures the link intensity between the unobserved heterogeneities of the two success probabilities, which allows us to statistically test for the link in a straightforward manner.
- Model 2—Model with joint random effect and unobserved heterogeneity
  We now extend Model 1 by incorporating an additional random effect term in $\mathrm{logit}(q_{lt})$. The extended model denoted by *Model 2* is given by
  (3) $$\begin{array}{ccc} \mathrm{logit}(p_{\ell t}) & = & \beta_{p0}^{\left(2\right)}+\beta_{p1}^{\left(2\right)}z_{\ell t1}+\ldots +\beta_{pk}^{\left(2\right)}z_{\ell tK}+a_{\ell }^{\left(2\right)}=z_{\ell t}\beta_{p}^{{}^{\prime }\left(2\right)}+a_{\ell }^{\left(2\right)},\\ \mathrm{logit}(q_{\ell t}) & = & \beta_{q0}^{\left(2\right)}+\beta_{q1}^{\left(2\right)}z_{\ell t1}+\ldots +\beta_{qk}^{\left(2\right)}z_{\ell tK}+\beta^{*(2)}a_{\ell }^{\left(2\right)}+\kappa_{\ell }\\ & = & z_{\ell t}\beta_{q}^{{}^{\prime }\left(2\right)}+\beta^{*(2)}a_{\ell }^{\left(2\right)}+\kappa_{\ell }, \end{array}$$
  where $a_{\ell }^{\left(2\right)}$ is regarded as the joint random effect, and $\kappa_{\ell }$ is the independent random effect of $\mathrm{logit}(q_{lt})$. We assume that $\kappa_{l}$ follows a standard normal distribution, as is the case for $a_{\ell }^{\left(2\right)}$. The implicit assumption behind Model 1 is that the joint random effect term $a_{\ell }^{\left(1\right)}$ is a single source of the unobserved heterogeneity for $\mathrm{logit}(p_{\ell t})$ and $\mathrm{logit}(q_{\ell t})$. However, this may not be the case depending on the data of various applications. In one extreme situation, in which $\mathrm{logit}(p_{\ell t})$ and $\mathrm{logit}(q_{\ell t})$ are completely independent after controlling for the effects of observed covariates, there will be no term in Model 1 that can reflect the unobserved heterogeneity of $\mathrm{logit}(q_{\ell t})$ because $\beta^{*}$ should be zero. In the most plausible scenario for which $\mathrm{logit}(p_{\ell t})$ and $\mathrm{logit}(q_{lt})$ are connected but not perfectly connected, we also need a term that can control for the unobserved heterogeneity in $\mathrm{logit}(q_{\ell t})$ independent of $a_{\ell }^{\left(1\right)}$. Therefore, Model 2 is designed to handle such cases.

## 3. Bayesian Inference

In this section, we present the Bayesian inference procedures for the proposed logistic regression models based on BVB distributions. Since the procedures regarding the prior and posterior distributions for Model 0, Model 1, and Model 2 are similar, for simplicity and illustrative purposes, we present the Bayesian inference for Model 1 only. We specify the prior and posterior distributions for the Bayesian analysis in Section 3.1 and describe the Bayesian computation procedures and algorithms in Section 3.2.

### 3.1. Prior and Posterior Distributions

Based on Model 1 in Equation (2) and the observed data $D_{\ell }$, ℓ=1,2,…,L, the likelihood function can be written as

(4) $$\begin{array}{ccc} & & p(\beta_{p}^{\left(1\right)},\beta_{q}^{\left(1\right)},\beta^{*(1)}|a^{\left(1\right)},z)\\ & \propto & \prod_{\ell =1}^{L}\prod_{t=1}^{T_{l}}\left[\frac{\exp\left\{\left(z_{\ell t}\beta_{p}^{{}^{\prime }\left(1\right)}+a_{\ell }^{\left(1\right)}\right)x_{\ell 1t}\right\}}{{\left\{1+\exp(z_{\ell t}\beta_{p}^{{}^{\prime }\left(1\right)}+a_{\ell }^{\left(1\right)})\right\}}^{m_{\ell t}}}\frac{\exp\left\{\left(z_{\ell t}\beta_{q}^{{}^{\prime }\left(1\right)}+\beta^{*(1)}a_{\ell }\right)x_{\ell 2t}\right\}}{{\left\{1+\exp(z_{\ell t}\beta_{q}^{{}^{\prime }\left(1\right)}+\beta^{*(1)}a_{\ell }^{\left(1\right)}\right\}}^{x_{\ell 1t}}}\right]\\ & & \times \prod_{\ell =1}^{L}\exp\left[-\frac{a_{\ell }^{2}}{2}\right]. \end{array}$$

To proceed with Bayesian estimation procedures, we specify prior distributions for the parameter vector $\Theta =(\beta_{pk}^{\left(1\right)},\beta_{qk}^{\left(1\right)},\beta^{*(1)})$ for k=0,1,⋯,K. We consider the following distributions under *independent a priori*:

(5) $$\begin{array}{c} \beta_{pk}^{\left(1\right)}∼N\left(0,\phi_{pk}^{2}\right),\;\beta_{qk}^{\left(1\right)}∼N\left(0,\phi_{qk}^{2}\right),\;\beta^{*(1)}∼N\left(0,\phi^{2}\right)\;\mathrm{for}\;k=0,1,\cdots ,K, \end{array}$$

where $\phi_{qk}^{2}$, $\phi_{pk}^{2}$ and $\phi^{2}$ are the variances of the normal prior distributions. Although these normal prior distributions provide a concise expression of the resultant conditional distribution, it is rather restrictive that the asymmetric distributions of the model parameters cannot be described. However, prior distributions other than normal distributions can be employed for the model parameters since our proposed Bayesian estimation algorithm in Section 3.2 can be applied to generic cases where the conditional distributions can be any arbitrary statistical distributions. Combining the likelihood function in Equation (4) and the joint prior distribution in (5) yields the joint posterior distribution

(6) $$\begin{array}{c} p(\beta_{p}^{\left(1\right)},\beta_{q}^{\left(1\right)},\beta^{*(1)}|D_{\ell },a_{\ell },\ell =1,2,\ldots ,L)\\ \;\propto \prod_{\ell =1}^{L}\prod_{t=1}^{T_{\ell }}\left[\frac{\exp\left\{\left(z_{\ell t}\beta_{p}^{{}^{\prime }\left(1\right)}+a_{\ell }\right)x_{\ell 1t}\right\}}{{\left\{1+\exp(z_{\ell t}\beta_{p}^{{}^{\prime }\left(1\right)}+a_{\ell })\right\}}^{m_{\ell t}}}\frac{\exp\left\{\left(z_{\ell t}\beta_{q}^{{}^{\prime }\left(1\right)}+\beta^{*(1)}a_{\ell }\right)x_{\ell 2t}\right\}}{{\left\{1+\exp(z_{\ell t}\beta_{q}^{{}^{\prime }\left(1\right)}+\beta^{*(1)}a_{\ell })\right\}}^{x_{\ell 1t}}}\right]\\ \;\times \prod_{\ell =1}^{L}\exp\left[-\frac{a_{\ell }^{2}}{2}\right]\times \prod_{k=1}^{K}\exp\left[-\frac{{\left(\beta_{pk}^{\left(1\right)}\right)}^{2}}{2\phi_{pk}^{2}}\right]\times \prod_{k=1}^{K}\exp\left[-\frac{{\left(\beta_{qk}^{\left(1\right)}\right)}^{2}}{2\phi_{qk}^{2}}\right]\times \exp\left[-\frac{\beta^{*(1)}{)}^{2}}{2\phi^{2}}\right]. \end{array}$$

To obtain the posterior estimates, we use the Metropolis–Hastings (MH) algorithm within Gibbs sampling. The full conditional distributions of parameters $\beta_{pk}^{\left(1\right)}$, $\beta_{qk}^{\left(1\right)}$, $\beta^{*(1)}$, and $a_{\ell }$ can be expressed as

$$\begin{array}{ccc} & & p(\beta_{pk}^{\left(1\right)}|\beta_{\left(-pk\right)}^{\left(1\right)},a_{\ell },D_{\ell },\ell =1,2,\ldots ,L)\\ & & \propto \prod_{\ell =1}^{L}\prod_{t=1}^{T_{\ell }}\left[\frac{\exp\left(\beta_{pk}^{\left(1\right)}z_{\ell tk}x_{\ell 1t}\right)}{{\left\{1+\exp(z_{\ell t}\beta_{p}^{{}^{\prime }\left(1\right)}+a_{\ell })\right\}}^{m_{\ell t}}}\right]\times \exp\left[-\frac{{\left(\beta_{pk}^{\left(1\right)}\right)}^{2}}{2\phi_{pk}^{2}}\right];\\ & & p(\beta_{qk}^{\left(1\right)}|\beta_{\left(-qk\right)}^{\left(1\right)},\beta^{*(1)},a_{\ell },D_{\ell },\ell =1,2,\ldots ,L)\\ & & \propto \prod_{\ell =1}^{L}\prod_{t=1}^{T_{l}}\left[\frac{\exp\left(\beta_{qk}^{\left(1\right)}z_{\ell tk}x_{\ell 2t}\right)}{{\left\{1+\exp(z_{\ell t}\beta_{q}^{{}^{\prime }\left(1\right)}+\beta^{*(1)}a_{\ell })\right\}}^{x_{\ell 1t}}}\right]\times \exp\left[-\frac{{\left(\beta_{qk}^{\left(1\right)}\right)}^{2}}{2\phi_{qk}^{2}}\right];\\ & & p(\beta^{*(1)}|\beta_{q}^{\left(1\right)},a_{\ell },D_{\ell },\ell =1,2,\ldots ,L)\\ & & \propto \prod_{\ell =1}^{L}\prod_{t=1}^{T_{l}}\left[\frac{\exp\left(\beta^{*(1)}a_{\ell }x_{\ell 2t}\right)}{{\left\{1+\exp(z_{\ell t}\beta_{q}^{{}^{\prime }\left(1\right)}+\beta^{*(1)}a_{\ell })\right\}}^{x_{\ell 1t}}}\right]\times \exp\left[-\frac{{\left(\beta^{*(1)}\right)}^{2}}{2\phi^{2}}\right];\\ & & p(a_{\ell }|\beta_{p}^{{}^{\prime }\left(1\right)},\beta_{q}^{{}^{\prime }\left(1\right)},\beta^{*(1)})\\ & & \propto \prod_{t=1}^{T}\left[\frac{\exp\left\{a_{\ell }\left(x_{\ell 1t}+\beta^{*(1)}x_{\ell 2t}\right)\right\}}{{\left\{1+\exp(z_{\ell t}\beta_{p}^{{}^{\prime }\left(1\right)}+a_{\ell })\right\}}^{m_{\ell t}}{\left\{1+\exp(z_{\ell t}\beta_{q}^{{}^{\prime }\left(1\right)}+\beta^{*(1)}a_{\ell })\right\}}^{x_{\ell 1t}}}\right]\\ & & \;\times \exp\left[-\frac{a_{\ell }^{2}}{2}\right], \end{array}$$

where $\beta_{\left(-pk\right)}^{\left(1\right)}$ and $\beta_{\left(-qk\right)}^{\left(1\right)}$ are *K* dimensional vectors excluding the *k*th component from the entire vector of covariates $\beta_{pk}^{\left(1\right)}$ and $\beta_{qk}^{\left(1\right)}$, respectively.

### 3.2. Markov Chain Monte Carlo (MCMC) Procedures

There is a considerable amount of work in the literature for generating random variates with Gibbs chains for which the full conditional density does not have a closed-form and hence, it is not easy to sample. The adaptive Metropolis–Hastings (AMH) algorithm is one of the commonly used algorithms that can be applied to construct proposal densities to closely approximate the target distribution. The AMH algorithm can be described as follows. Consider that there are *d* parameters and the normal distribution is used as a proposal density. The current value of a parameter is used as the mean of the normal distribution and an arbitrary value can be used as the variance of the normal distribution. The mean and the variance of the normal distribution are updated in every step using the adjusted parameters and the empirical variance. Suppose we are interested in updating parameter θ in the h+1-th step given the initial value of the parameter $\theta^{\left(0\right)}$ and the values of the parameter in the first *h* steps as $\theta^{\left(1\right)}$, $\theta^{\left(2\right)}$, $\ldots ,\theta^{\left(h\right)}$. The candidate value $\theta^{*}$ can be generated from a normal distribution with mean $\theta^{\left(h\right)}$ and variance $V^{\left(h\right)}$, where

(7) $$\begin{array}{c} V^{\left(h\right)}=\left\{\begin{array}{c} V^{\left(0\right)},\;\mathrm{if}\;h=0,\\ \delta \left[\mathrm{Var}\left(\theta^{\left(0\right)},\ldots ,\theta^{\left(h-1\right)}\right)+\omega \right],\;\mathrm{if}\;h>0. \end{array}\right. \end{array}$$

Here, $V^{\left(0\right)}$ is an initial (could be arbitrary) variance of the proposal distribution of parameter θ and δ is the adjusting coefficient. We use the value of $\delta =2.4/\sqrt{d}$ with *d* being the dimension of the parameter space to maintain the acceptance rate of the candidate value $\theta^{*}$. The value of δ ensures that the optimal value of the acceptance rate is about 0.44 [13]. Moreover, ω should be assigned as a very small value to prevent the variance in (7) from being zero.

On the other hand, we apply the Independent Metropolis–Hastings (IMH) algorithm for the random effects $a_{\ell }$ to extract the candidate value $\theta^{*}$ from a normal distribution regardless of the current value $\theta^{\left(t\right)}$. Based on our settings, the IMH algorithm for applying random effects can be described as follows:

**Step** **1.** Given the current estimate $\theta^{\left(h\right)}$, in the *h*-th iteration, generate $\theta^{*}$ from a standard normal distribution
  $$\begin{array}{ccc} \theta^{*} & ∼ & N\left(0,1\right)=\pi \left(\theta^{*}\right). \end{array}$$
**Step** **2.** Compute the ratio α, composed of the full conditional distribution *p* and the prior densities π.
  $$\begin{array}{ccc} \alpha (\theta^{\left(h\right)},\theta^{*}) & = & \frac{p\left(\theta^{*}\right)\pi \left(\theta^{\left(h\right)}\right)}{p\left(\theta^{\left(h\right)}\right)\pi \left(\theta^{*}\right)}. \end{array}$$
**Step** **3.** Draw u∼Uniform(0,1). If $u\leq \alpha (\theta^{\left(h\right)},\theta^{*})$, then $\theta^{\left(h+1\right)}=\theta^{*}$. Otherwise $\theta^{\left(h+1\right)}=\theta^{\left(h\right)}$.

Note that the candidate generating distribution in Step 1 is different from the original target distributions of the model parameters. According to the acceptance probability α in Step 2, the discrepancy between these two distributions is corrected by randomly accepting or rejecting the random samples from the candidate distribution. For more details of the Metropolis–Hastings algorithm, one may refer to [14]. Repeat Steps 1–3 H=6000 times to obtain the sequences of estimates $(\theta^{\left(1\right)},\theta^{\left(2\right)},\ldots ,\theta^{\left(H\right)}$). Consider the first B=1000 estimates as burn-in, the posterior mean of θ, denoted as $\tilde{\theta }$, based on the IMH algorithm can be obtained as

$$\begin{array}{c} \tilde{\theta }=\frac{1}{H-B}\sum_{h=B+1}^{H}\theta^{\left(h\right)}. \end{array}$$

Based on the marginal posterior distributions of the parameters, one can construct a highest posterior density (HPD) interval for each of the model parameters. Let π(θ|x) be the posterior density for θ. A 100(1−γ)% HPD credible set *C* is a subset of Θ which satisfies C={θ∈Θ|π(θ|x)≥k(γ)}, where k(γ) is the largest number such that Pr(θ∈C|x)≥1−γ. Since each of the marginal posterior distributions does not have a closed form, we use the sequences of estimates in Gibbs chains described above to find HPD intervals with a given nominal level of 100(1−γ)% [15,16].

## 4. Practical Data Analysis

For sports statistics, statistical analysis of baseball data has become more important in professional baseball. In the past two decades, there have been a substantial number of studies on measuring the offensive abilities of MLB players see, for example [17,18,19,20,21]. Readers who are interested in the field of sports statistics may refer to the book by [22]. In this practical data analysis, we consider a dataset on MLB players with several covariates commonly used to evaluate batters’ hitting performances and apply the proposed logistic regression model based on the BVB distribution.

Here are the definitions of the variables considered in this analysis:

- Win Probability Added (WPA): The percent change in a team’s chances of winning from one game to the next;
- Center percentage (Cent%): The percentage of balls in play that were hit to center fields by batters;
- Pull percentage (Pull%): The percentage of balls in play that were pulled by hitters;
- Opposite percentage (Oppo%): The percentage of balls in play that were hit to opposite fields by batters;
- BABIP (Batting Average on Balls in Play): A statistic indicating how often a ball in play goes for a hit;
- Walk to strikeout ratio (BB/K): A batting ratio that shows the ratio of walks for each strikeout. The higher the ratio, the better the performance;
- Home run to fly ball ratio (HR/FB): The ratio of how many home runs are hit against a pitcher for every fly ball he/she allows;
- Line drive percentage (LD%): The percentage of balls hit into the field of play that are characterized as line drives;
- Ground ball percentage (GB%): The percentage of batted balls hit as ground balls against a pitcher;
- Fly ball percentage (FB%): The percentage of fly balls hit into the field of play.

There are 30 teams in MLB among which two players from each team are selected. The regular season of MLB starts in early April and finishes at the end of September, during which 162 games are played. We used the data for the year 2021, which can be collected online: https://www.fangraphs.com/ (accessed on 19 July 2022). In the dataset used here, there are 60 players (i.e., L=60) measured at T=6 time points (monthly from April to September). Parameter *p* denotes the batting average, and parameter *q* denotes the proportion of extra-base hits (two-base hits, three-base hits, and home runs) out of the total hits. In this empirical analysis, we use the demeaned covariates by their time series means so that the intercept coefficients or random effects can be directly translated into a play’s performance.

First, we compare the results with the frequentist approach under Model 0 in (1). The MLEs can be obtained by maximizing the likelihood function in Equation (4) without the term $a_{\ell }$. Let $\left({\hat{\beta }}_{p}^{\left(0\right)},{\hat{\beta }}_{q}^{\left(0\right)}\right)$ denote the MLEs of the parameter vectors $\left(\beta_{p}^{\left(0\right)},\beta_{q}^{\left(0\right)}\right)$. The asymptotic distribution of $\left({\hat{\beta }}_{p}^{\left(0\right)},{\hat{\beta }}_{q}^{\left(0\right)}\right)$ is 2(K+1)-variate multivariate normal with mean vector ($\beta_{p}^{\left(0\right)}$, $\beta_{q}^{\left(0\right)}$) and variance-covariance matrix $V({\hat{\beta }}_{p}^{\left(0\right)},{\hat{\beta }}_{q}^{\left(0\right)})$, where

$$\begin{array}{c} V\left({\hat{\beta }}_{p}^{\left(0\right)},{\hat{\beta }}_{q}^{\left(0\right)}\right)={\left[v_{kk^{\prime }}\right]}_{2(K+1)\times 2(K+1)}=I^{-1}\left({\hat{\beta }}_{p}^{\left(0\right)},{\hat{\beta }}_{q}^{\left(0\right)}\right). \end{array}$$

Here, $I^{-1}\left({\hat{\beta }}_{p}^{\left(0\right)},{\hat{\beta }}_{q}^{\left(0\right)}\right)$ is the inverse of the observed Fisher information matrix. We can test the significance of the regression parameter, i.e., testing the hypothesis $H_{0}:\beta_{pk}^{\left(0\right)}=0$ vs. $H_{a}:\beta_{pk}^{\left(0\right)}\neq 0$, or $H_{0}:\beta_{qk}^{\left(0\right)}=0$ vs. $H_{a}:\beta_{qk}^{\left(0\right)}\neq 0$, k=0,1,2,…,K, based on the asymptotic properties of the MLEs. For example, to test $H_{0}:\beta_{pk}^{\left(0\right)}=0$ vs. $H_{a}:\beta_{pk}^{\left(0\right)}\neq 0$, we consider the test statistic ${\hat{\beta }}_{pk}^{\left(0\right)}/\sqrt{v_{kk}}$ with *p*-value $\mathrm{Pr}(Z>|{\hat{\beta }}_{pk}^{\left(0\right)}/\sqrt{v_{kk}}|)$, where *Z* is the standard normal random variable. In summary, we consider the following covariates for modeling probabilities *p* and *q*:

- Variables influencing both *p* and *q*: WPA, Cent%;
- Variables influencing *p* only: BABIP, BB/K, LD%, GB%, Oppo%;
- Variables influencing *q* only: FB%, HR/FB, Pull%.

We define $z_{1t}=\mathrm{WPA}$, $z_{2t}=\mathrm{Cent}\%$, $z_{3t}=\mathrm{BABIP}$, $z_{4t}=\mathrm{BB}/K$, $z_{5t}=\mathrm{LD}\%$, $z_{6t}=\mathrm{GB}\%$, $z_{7t}=\mathrm{Oppo}\%$, $z_{8t}=\mathrm{FB}\%$, $z_{9t}=\mathrm{HR}/\mathrm{FB}$, $z_{10t}=\mathrm{Pull}\%$ with $\beta_{3q}^{\left(0\right)}=\beta_{4q}^{\left(0\right)}=\beta_{5q}^{\left(0\right)}=\beta_{6q}^{\left(0\right)}=\beta_{7q}^{\left(0\right)}=0$ and $\beta_{8p}^{\left(0\right)}=\beta_{9p}^{\left(0\right)}=\beta_{10p}^{\left(0\right)}=0$.

We also applied the proposed Bayesian estimation method presented in Section 3 based on Model 0. Regarding the hyperparameters $\phi_{pk}^{2}$, $\phi_{qk}^{2}$, and $\phi^{2}$ in the normal priors, we set the variances as 100, which leads to vague or weakly informative priors that reflect the lack of prior knowledge of the model parameters. Vague or weakly informative priors have been extensively used in [23,24]. To assess the robustness of the results for different variances in the normal priors, we conducted the Bayesian inference under other variances in the normal priors and compared the results (the results are not shown here for the sake of brevity). We found that the results from Bayesian inference are fairly robust to the changes on the hyperparameters.

The Bayes estimates are obtained from 6000 MCMC iterations with 1000 burn-in iterations. In Table 1, we provide the MLEs, the Bayesian posterior means, 95% HPD intervals, and *p*-values for testing if each coefficient is zero based on the procedures described above.

From Table 1, the frequentist and Bayesian approaches are congruent. Specifically, both approaches yield estimates of the parameters that are close to each other. Moreover, the parameters that have *p*-values based on MLEs smaller than 5% are also those for which the corresponding 95% HPD interval does not contain zero.

From variable selection perspectives, WPA turns out to be significant for *p* while it was barely not for *q*. Notice that the estimates of coefficients associated with WPA have different signs for *p* and *q*, which implies that WPA has a positive impact on the “batting average” while WPA has a negative impact on producing more extra-base hits when keeping other variables fixed. This is within our expectation, since Model 0 consists of two independent logit functions. The factor BABIP yields a significant *p*-value among all the variables in conjunction with the batting average *p*. We notice that BABIP is one of the important factors in modern baseball to assess batters’ performance [25,26]. The covariate BB/K reveals a decently significant result for modeling the batting average. Both FB% and HR/FB show significant impacts on predicting *q* with parameter estimates that have positive signs.

**Figure 1:**
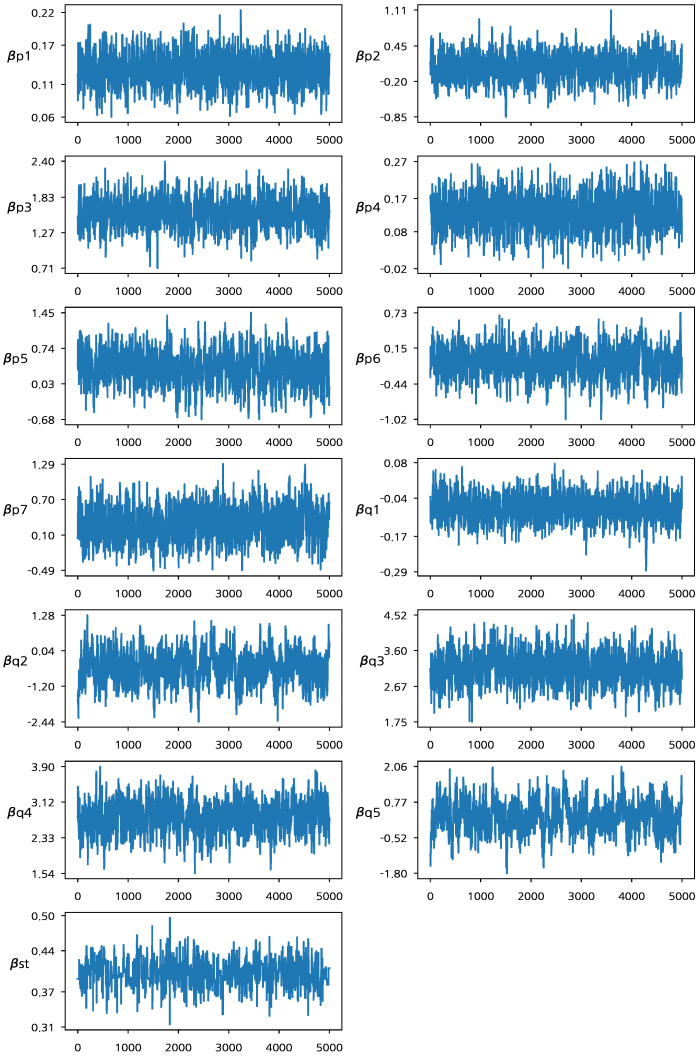
Traceplots of MCMC samples based on Model 1.

As discussed in Section 2, Model 0 does not consider random effects, therefore, we apply Model 1 using the Bayesian approach for the baseball data. Table 2 presents the parameter estimates (posterior mean), the standard deviations (SD) of the estimates, and the 95% HPD interval for Model 1 in (2). The Bayes estimates are obtained from 6000 MCMC iterations with 1000 burn-in iterations. The assessment of convergence of the Gibbs sampling is carried out based on the methodology proposed by [27]. Figure 1 presents the trace plots of the MCMC samples. From Figure 1, we can see that the MCMC chain of the Bayesian estimation algorithm has converged to the target posterior distribution after 1000 MCMC iterations. When fitting Model 1, we excluded the two intercept terms $\beta_{p0}^{\left(1\right)}$ and $\beta_{q0}^{\left(1\right)}$ in order for random effects to capture both the average performances and variabilities between players [28,29]. In addition, the exclusion of the intercept terms improves the convergence of the MCMC algorithm (We note that a player’s average performance can be captured by both intercept and random effect terms. Thus, the MCMC samples of the two terms are strongly correlated if they are drawn from their respective conditional posterior distributions which significantly affect each other. This makes the convergence of the proposed algorithm slow). The posterior mean for $\beta^{*(1)}$ is 0.402, and the corresponding 95% HPD interval is (0.3562, 0.4482), which does not contain zero. This reflects that there is a meaningful connection between the two logit functions associated with the joint random effects and provides the basis to claim that the two success probabilities should be jointly investigated to evaluate a player’s underlying performance.

Compared to the results based on Model 0 in Table 1, the signs of the estimates of regression parameters are the same for all the parameters, though there are slight changes in magnitude. Comparing the results based on Model 0 and Model 1 provides us with an important piece of information on the property of the random effects. The variation in the unobserved heterogeneity may or may not be correlated with the covariates of the BVB regression model.

If variation in the unobserved heterogeneity and the covariates are correlated, then Model 0 that does not contain the individual effects will produce biased estimates which are different from those estimates of Model 1 with the random effect terms. Note that Model 1 directly estimates the random effects, which always produces unbiased estimates regardless of the relation between the unobserved heterogeneity and the covariates. On the other hand, Model 0 produces unbiased estimates only when there is no correlation between the unobserved heterogeneity and the covariates. Thus, the closeness of the parameter estimates based on Model 0 and Model 1 provides suggestive evidence that the unobserved heterogeneity is not associated with any of the covariates included in the regression model.

**Figure 2:**
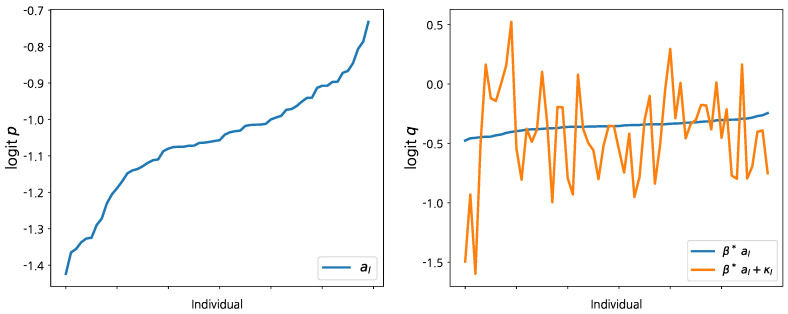
The posterior means for $a_{l}^{\left(2\right)}$ under Model 2 in conjunction with success probability *p* (**left panel**), and the posterior means for two different random effects based on the player labels according to $a_{\ell }^{\left(2\right)}$ (**right panel**).

After considering Model 1, we also apply Model 2 to the baseball dataset. The empirical results of Model 2 are reported in Figure 2 and Table 3. The MH algorithm with slight modifications is used to incorporate $\kappa_{\ell }$ in the model. As in fitting Model 1, we fix $\beta_{p0}^{\left(2\right)}=\beta_{q0}^{\left(2\right)}=0$ and do not include these terms in the computation procedure to avoid slow convergence (In Model 2, the intercept coefficients are redundant given the two random effect terms). We note that Model 2 requires a larger number of iterations for the convergence in the computation due to the additional random effect term $\kappa_{\ell }$ (The traceplot of MCMC samples for Model 2 is not reported in this section for the sake of brevity. The traceplot shows that all model parameters converge to their stationary posterior distributions).

The left panel of Figure 2 displays the posterior means for $a_{\ell }^{\left(2\right)}$, which is the unobserved heterogeneity in conjunction with success probability *p*. To get a meaningful interpretation, the estimates of $\left\{a_{1}^{\left(2\right)},a_{2}^{\left(2\right)},\ldots ,a_{L}^{\left(2\right)}\right\}$ are sorted in ascending order and we label each MLB player accordingly. The first player’s average success probability (batting average) *p* over the full six-month sample period is estimated as exp{−1.4}/[1+exp{−1.4}]≈0.19. (Recall that we use demeaned covariates. Thus, the estimated random effects can be interpreted as the average performance of a player.) The last player’s average success probability *p* can be estimated as exp{−0.7}/[1+exp{−0.7}]≈0.33. Hence, the average success probabilities of the other MLB players considered in this dataset are in between 0.19 and 0.33 based on this analysis. The right panel of Figure 2 compares the random effects of the two logit functions based on the player labels according to $a_{\ell }^{\left(2\right)}$. Although the posterior estimates in the second panel are noisy, a similar pattern with $a_{\ell }^{\left(2\right)}$ is hidden in the posterior means of $\beta^{*(2)}a_{\ell }^{\left(2\right)}+\kappa_{\ell }$, which represent the unobserved heterogeneity of success probability *q*. However, this is only suggestive evidence because the posterior estimates of $\kappa_{\ell }$ vary significantly across players.

To obtain more concrete empirical evidence on the link between *p* and *q*, we check the posterior estimate of $\beta^{*(2)}$ in the last row of Table 3. Although the confidence interval for $\beta^{*}$ is wider than that of Model 1, the sign of the estimate is still positive and the 95% HPD interval does not contain zero. From these results, we claim that the two success probabilities are inter-connected even after controlling for the effects of the commonly used covariates. The other Bayes estimates of the model parameters in Table 3 confirm that the estimated marginal effects are consistent across different model specifications.

## 5. Monte Carlo Simulation Studies

To empirically demonstrate the performance of the proposed regression model and the Bayesian estimation method, we perform Monte Carlo simulation studies with different settings. We generate 200 sets of data with m=100, L=30, and T=6 based on Model 0 and Model 1. We also consider Model 2 in the simulation study, but the simulation results for Model 2 are not presented here, as the algorithm for estimating the parameters in Model 2 is a simple extension of the algorithm for Model 1. For each dataset, we estimate the corresponding model parameters in the two models with the MH algorithm described in Section 3. For Model 1, the two intercept terms $\beta_{p0}^{\left(1\right)}$ and $\beta_{q0}^{\left(1\right)}$ are set to be zero and they are not estimated to increase the speed of the convergence of the estimation algorithm. For Model 0, the true values of the model parameters are set to be $\left\{\beta_{p0}^{\left(0\right)}=1,\beta_{p1}^{\left(0\right)}=-1,\beta_{p2}^{\left(0\right)}=2,\beta_{q0}^{\left(0\right)}=-1,\beta_{q1}^{\left(0\right)}=1,\beta_{q2}^{\left(0\right)}=-2\right\}$. For Model 1, the true values of the model parameters are set to be $\left\{\beta_{p1}^{\left(1\right)}=-1,\beta_{p2}^{\left(1\right)}=2,\beta_{q1}^{\left(1\right)}=1,\beta_{q2}^{\left(1\right)}=-2,\beta^{*(1)}=1\right\}$. We have considered other sets of true parameters, and the simulation results lead to qualitatively similar conclusions that the MH algorithm works well. Therefore, the simulation results for other settings are omitted in this paper. The random effect term $a_{\ell }^{\left(1\right)}$ is generated from the standard normal distribution, i.e., $a_{\ell }^{\left(1\right)}∼N\left(0,1\right)$.

For each simulated dataset, we compute the posterior means and HPD intervals of the model parameters. After collecting the posterior estimates, we evaluate the performance of the point estimates based on the average posterior mean, the bias, and the mean squared error (MSE); and evaluate the performance of the interval estimates based on the coverage probability (CP), and average width (AW). The CP represents the proportion that the 95% HPD interval contains the true value. The total and burn-in MCMC iterations of the Bayesian estimation are set to be 6000 and 1000, respectively.

Table 4 presents the simulation results of Model 0. The simulated average posterior means, biases, and MSEs show that the proposed Bayesian estimation procedure performs well for point estimation. The simulated CPs show that the Bayesian 95% credible intervals control the coverage probabilities at or above the nominal level in most cases. Moreover, the simulated AWs show that the 95% credible intervals provides reasonable interval estimates. Overall, the proposed Bayesian estimation procedure with the MH algorithm can accurately estimate the model parameters for Model 0.

The simulation results of Model 1 are presented in Table 5. Similar conclusions about the performance of the point and interval estimates based on the proposed Bayesian approach can be obtained as they were for Model 0. The major difference between Model 0 and Model 1 is that the amount of uncertainty in the estimation process is larger in Model 1 due to the inclusion of random effects. The increase in uncertainty explains the larger simulated MSEs and AWs of the estimates in Model 1 compared with those in Model 0. These results are a natural consequence of introducing joint random effects to the logit functions. The results in Table 5 show that the parameter $\beta^{*(1)}$ that plays a special role in our model can be precisely estimated by the proposed Bayesian approach.

## 6. Concluding Remarks

This paper proposes a new regression model based on a bivariate binomial (BVB) distribution that is applicable when two success probabilities are inter-connected. The main feature of the proposed model is that common random effects for the two success probabilities are considered. By employing the conventional Markov chain Monte Carlo method, we explicitly estimate the common random effects along with a link intensity parameter representing how strongly the pair of the two probabilities are linked via the joint unobserved heterogeneity. For an empirical illustration, the proposed BVB regression models are applied to the data of 60 Major League Baseball (MLB) batters. The Bayes estimates for the models suggest that the two success probabilities assessing each player’s performance are closely related even after controlling for the player’s observed characteristics.

Classical estimates for model parameters of a panel regression model could be biased if an unobserved heterogeneity is not adequately handled in the estimation procedures. By considering the common random effects and directly estimating them, we show that the estimated marginal effects of the observed covariates are robust to potential model misspecifications that can arise when random effects are ignored. Last but not least, we extend the proposed model to incorporate common and independent random effects in two logit functions. From empirical findings, we assure that both types of random effects play an important role in evaluating the performances of players. The proposed BVB regression model can be used to study the possibility that unobserved heterogeneities are inter-connected under two logit functions. Another interesting extension would be considering common time fixed effects. Investigations in these directions are in progress, and we hope to report the results in a future paper.

## Figures and Tables

**Table 1:** Results for maximum likelihood estimates and Bayes estimates on Model 0.

Parameter	MLE	Posterior Mean	95% HPD Interval	*p*-Value
$\beta_{p0}^{\left(0\right)}$	−1.0362	−1.0376	(−1.0618,−1.0135)	<0.0001
$\beta_{p1}^{\left(0\right)}$	0.1334	0.1330	(0.0847,0.1814)	<0.0001
$\beta_{p2}^{\left(0\right)}$	0.0609	0.0663	(−0.4381,0.5708)	0.4032
$\beta_{p3}^{\left(0\right)}$	1.6197	1.6186	(1.1803,2.0569)	<0.0001
$\beta_{p4}^{\left(0\right)}$	0.1327	0.1339	(0.0436,0.2242)	0.0024
$\beta_{p5}^{\left(0\right)}$	0.2536	0.2796	(−0.3921,0.9514)	0.2247
$\beta_{p6}^{\left(0\right)}$	−0.0994	−0.0826	(−0.5843,0.4190)	0.3465
$\beta_{p7}^{\left(0\right)}$	−0.2794	0.2993	(−0.2889,0.8878)	0.1684
$\beta_{q0}^{\left(0\right)}$	−0.4136	−0.4145	(−0.4591,−0.3701)	<0.0001
$\beta_{q1}^{\left(0\right)}$	−0.0729	−0.0713	(−0.1575,0.0147)	0.0609
$\beta_{q2}^{\left(0\right)}$	−0.5332	−0.5551	(−1.5886,0.4782)	0.1564
$\beta_{q8}^{\left(0\right)}$	3.1443	3.1411	(2.3109,3.9712)	<0.0001
$\beta_{q9}^{\left(0\right)}$	2.7858	2.7795	(2.1279,3.4311)	<0.0001
$\beta_{q10}^{\left(0\right)}$	0.2409	0.1981	(−0.8541,1.2504)	0.3229

**Table 2:** Bayes estimates and related results for Model 1.

Parameter	Posterior Mean	SD	95% HPD Interval
$\beta_{p1}^{\left(1\right)}$	0.1331	0.0247	(0.0847, 0.1815)
$\beta_{p2}^{\left(1\right)}$	0.0594	0.2452	(−0.4212, 0.54)
$\beta_{p3}^{\left(1\right)}$	1.5778	0.2335	(1.1202, 2.0355)
$\beta_{p4}^{\left(1\right)}$	0.1320	0.0467	(0.0404, 0.2236)
$\beta_{p5}^{\left(1\right)}$	0.3470	0.3343	(−0.3082, 1.0022)
$\beta_{p6}^{\left(1\right)}$	−0.0776	0.2540	(−0.5754, 0.4202)
$\beta_{p7}^{\left(1\right)}$	0.2890	0.2830	(−0.2657, 0.8436)
$\beta_{q1}^{\left(1\right)}$	−0.0727	0.0442	(−0.1593, 0.0139)
$\beta_{q2}^{\left(1\right)}$	−0.5374	0.5697	(−1.6539, 0.5792)
$\beta_{q8}^{\left(1\right)}$	3.1403	0.4117	(2.3334, 3.9471)
$\beta_{q9}^{\left(1\right)}$	2.7825	0.3460	(2.1044, 3.4606)
$\beta_{q10}^{\left(1\right)}$	0.2389	0.5597	(−0.8581, 1.3359)
$\beta^{*(1)}$	0.4022	0.0235	(0.3562, 0.4482)

**Table 3:** Bayes estimates and related results for Model 2.

Parameter	Posterior Mean	SD	95% HPD Interval
$\beta_{p1}^{\left(2\right)}$	0.1347	0.0253	(0.085, 0.1843)
$\beta_{p2}^{\left(2\right)}$	0.0657	0.2553	(−0.4347, 0.5661)
$\beta_{p3}^{\left(2\right)}$	1.6181	0.2349	(1.1576, 2.0785)
$\beta_{p4}^{\left(2\right)}$	0.1332	0.0454	(0.0441, 0.2223)
$\beta_{p5}^{\left(2\right)}$	0.3275	0.3317	(−0.3227, 0.9777)
$\beta_{p6}^{\left(2\right)}$	−0.0688	0.2603	(−0.5789, 0.4413)
$\beta_{p7}^{\left(2\right)}$	0.3164	0.2902	(−0.2524, 0.8852)
$\beta_{q1}^{\left(2\right)}$	−0.0599	0.0464	(−0.1509, 0.0312)
$\beta_{q2}^{\left(2\right)}$	−0.5264	0.5452	(−1.595, 0.5421)
$\beta_{q8}^{\left(2\right)}$	3.2221	0.3872	(2.4631, 3.9811)
$\beta_{q9}^{\left(2\right)}$	2.7660	0.3507	(2.0787, 3.4533)
$\beta_{q10}^{\left(2\right)}$	0.3569	0.5355	(−0.6927, 1.4064)
$\beta^{*(2)}$	0.3344	0.1131	(0.1127, 0.5561)

**Table 4:** Simulated biases, MSEs for point estimation, coverage probabilities (CP) and average widths (AW) of 95% credible intervals of all parameters for sample sizes of m=100 and L=30 for 6 time points with 200 replications in Model 0.

Parameter	True Value	Posterior Mean	Bias	MSE	CP	AW
$\beta_{p0}^{\left(0\right)}$	1	1.0007	0.0007	0.0006	0.9750	0.0700
$\beta_{p1}^{\left(0\right)}$	−1	−1.0031	−0.0031	0.0064	0.9700	0.2304
$\beta_{p2}^{\left(0\right)}$	2	2.0030	0.0030	0.0085	0.9400	0.2427
$\beta_{q0}^{\left(0\right)}$	−1	−1.0002	−0.0002	0.0009	0.9500	0.0829
$\beta_{q1}^{\left(0\right)}$	1	0.9984	−0.0016	0.0096	0.9550	0.2755
$\beta_{q2}^{\left(0\right)}$	−2	−2.0074	−0.0074	0.0110	0.9350	0.2880

**Table 5:** Simulated biases, MSEs for point estimation, coverage probabilities (CP) and average widths (AW) of 95% credible intervals of all parameters for sample sizes m=100 and L=30 for 6 time points with 200 replications for Model 1.

Parameter	True Value	Posterior Mean	Bias	MSE	CP	AW
$\beta_{p1}^{\left(1\right)}$	−1	−1.0223	−0.0223	0.0161	0.9375	0.3376
$\beta_{p2}^{\left(1\right)}$	2	1.9982	−0.0018	0.0155	0.9625	0.3577
$\beta_{q1}^{\left(1\right)}$	1	0.9900	−0.0100	0.0275	0.9500	0.4707
$\beta_{q2}^{\left(1\right)}$	−2	−1.9895	0.0105	0.0335	0.9625	0.4960
$\beta^{*(1)}$	1	1.0017	0.0017	0.0055	0.9500	0.2050

## Data Availability

Not applicable.
